# HIV Information Acquisition and Use Among Young Black Men Who Have Sex With Men Who Use the Internet: Mixed Methods Study

**DOI:** 10.2196/22986

**Published:** 2021-05-07

**Authors:** Megan Threats, Keosha Bond

**Affiliations:** 1 School of Communication and Information Rutgers University New Brunswick, NJ United States; 2 Yale School of Public Health Center for Interdisciplinary Research on AIDS New Haven, CT United States; 3 Department of Community Health and Social Medicine City University of New York School of Medicine New York, NY United States

**Keywords:** HIV, health information behavior, eHealth, mHealth social media, consumer health informatics, mobile phones, sexual and gender minorities, African Americans, young adults, mixed methods

## Abstract

**Background:**

HIV disproportionately affects young Black men who have sex with men (YBMSM) in the United States. eHealth holds potential for supporting linkage and engagement in HIV prevention and care and the delivery of HIV information to YBMSM.

**Objective:**

This study aims to investigate HIV information acquisition and use among YBMSM who use the internet.

**Methods:**

A web-based self-administered survey and semistructured interviews were conducted. The survey findings informed the development of the interview guide. Descriptive statistics were used to characterize the survey sample, and interview data were analyzed thematically using modified grounded theory methodologies.

**Results:**

Among the internet sample (N=83), the average age was 29.2 (SD 3.5) years, 41% (n=34) of participants self-reported living with HIV, 43% (n=36) were HIV-negative, and 15% (n=13) were unsure of their HIV status. Most participants (n=79, 95%) acquired HIV information through the internet while using a mobile phone. Web-based HIV information was intentionally sought from consumer health information websites (n=31, 37%), government health information websites (n=25, 30%), and social media (n=14, 17%). Most men incidentally acquired HIV information via advertisements on social media sites and geospatial dating apps (n=54, 65%), posts on social media sites from their web-based social ties (n=44, 53%), and advertisements while browsing the internet (n=40, 48%). Although the internet is the top source of HIV information, health care providers were the most preferred (n=42, 50%) and trusted (n=80, 96%) source of HIV information. HIV information was used to facilitate the use of HIV prevention and care services. The qualitative sample included YBMSM across a range of ages and at different points of engagement in HIV prevention and care. Qualitative findings included the importance of the internet as a primary source of HIV information. The internet was used because of its ease of accessibility, because of its ability to maintain anonymity while searching for sensitive information, and to mitigate intersecting stigmas in health care settings. Participants used HIV information to assess their risk for HIV and AIDS, support their skill building for HIV prevention, inform patient–doctor communication, and learn about HIV prevention and treatment options. Men expressed concerns about their diminishing access to online spaces for HIV information exchange among YBMSM because of censorship policies on social media sites and the *stigmatizing* framing and tone of mass media HIV-prevention advertisements encountered while using the internet.

**Conclusions:**

YBMSM in this sample had high utilization of eHealth for HIV information acquisition and use but diminished access to their preferred and most trusted source of HIV information: health care providers. Future eHealth-based HIV interventions culturally tailored for YBMSM should aim to reduce intersectional stigma at the point of care and support patient–provider communication. The findings demonstrate the need for community-informed, culturally tailored HIV messaging and online spaces for informational support exchange among YBMSM.

## Introduction

### Background

Young Black men who have sex with men (YBMSM) are disproportionately affected by HIV in the United States, accounting for more HIV diagnoses than men who have sex with men (MSM) of other ethnoracial groups [1,2]. Black MSM have a 50% chance of acquiring HIV in their lifetime, and if the current incidence rates persist, 40% of Black MSM will be diagnosed with HIV by the age of 30 years [3,4]. YBMSM experience disparities across every stage of the HIV prevention and care continuum [5-8]. Compared with MSM of other ethnicities, YBMSM are less likely to have knowledge of their HIV status and are more likely to be infected, yet unaware [1,6]. Biomedical advancements in HIV prevention and treatment, such as pre-exposure prophylaxis (PrEP), provide an opportunity to reduce HIV transmission and related disparities, but awareness and adoption of these advancements remain the lowest among YBMSM [9-14].

### Related Literature

HIV-related information is a critical resource for people living with HIV (PLWH) and those at high risk for HIV. Assessing and managing the risk of acquiring or transmitting HIV requires the successful acquisition and application of accurate HIV information. As possessing HIV information does not automatically lead to health behavior change, individuals must be informed before they are motivated and equipped with the skills to perform HIV risk-reduction behaviors [15,16]. Improving our knowledge of how YBMSM acquire and use HIV-related information outside of planned interventions will aid in efforts to disseminate information and messages among this group, which is at an elevated risk for HIV.

There is a growing body of literature that examines health information behavior (eg, health information acquisition, sharing, management, and use) within the domain of HIV [17-20]. Much of the literature examining HIV-related health information behavior has been explored among samples consisting primarily of PLWH, heterosexual youth, and individuals living outside of the United States [21-27]. Consequently, such findings are not generalizable to YBMSM. For instance, because the HIV epidemic disproportionately affects YBMSM, heterosexual young adults often seek information about contraception (eg, pregnancy prevention) and less information about HIV [28-30]. Furthermore, previous research examining HIV information acquisition among Black MSM was conducted at a time when the internet was not as accessible via wireless connection and mobile devices [31].

There is an opportunity to leverage eHealth modalities (eg, the internet, mobile technologies, social media, video games, geospatial networking apps, and text messaging) to provide access to accurate HIV information and support its use among YBMSM [32-36]. Although there are several technology-enabled HIV interventions that provide access to credible HIV information and aim to improve HIV-related outcomes among YBMSM, most are specifically for YBMSM living with HIV [37-40]. Few eHealth or mobile health (mHealth) HIV interventions are inclusive of YBMSM of varying HIV status [41-46]. To inform the design of future eHealth and mHealth interventions that provide HIV-related information and encourage its application among YBMSM, we need a better understanding of how YBMSM currently use these technologies to access and interact with HIV information and services outside of planned interventions. This paper describes a part of a larger study on HIV-related information behaviors of YBMSM and factors affecting their engagement along the HIV prevention and care continuum [47]. This study aims to explore HIV information acquisition and use among YBMSM who use the internet.

## Methods

### Mixed Methods Study Design

A sequential, explanatory mixed methods design guided this study [48]. During the first phase of the study, a convenience sample of 83 YBMSM completed a web-based, self-administered survey. The survey data were then analyzed to help inform the development of the interview guide. In the second phase of the study, semistructured interviews were conducted among a subsample (n=22) of survey respondents to help elaborate on the survey findings and provide new insights. The survey and interview data were integrated or *mixed* during both the collection and analysis of data. The results from the quantitative and qualitative data analyses were triangulated during interpretation.

### Participants and Recruitment

A convenience sample of 83 YBMSM was recruited from December 18, 2018, to May 28, 2019, through a variety of venues such as HIV or sexually transmitted infection (STI) clinics, AIDS Service Organizations, a convenience sample from the UNC Center for AIDS Research, social media sites, geospatial dating apps, bars or clubs, lesbian, gay, bisexual, trans, and queer (LGBTQ) organizations, historically Black colleges and universities, mobile instant messaging apps (GroupMe and WhatsApp), and public postings. The eligibility criteria included the following: self-identification as Black or African American; identification as gay, bisexual, same-gender loving, or a man who has had sex with other men; being aged between 18 and 34 years; residing in the state of North Carolina; willing and able to provide informed consent; and being able to read, write, and speak English. The study received ethical approval from the institutional review board of the University of North Carolina at Chapel Hill. A certificate of confidentiality was obtained from the National Institutes of Health for the study.

### Part I: Internet Survey

The survey adapted questions from 3 previous surveys: the National Cancer Institute’s Health Information National Trends Survey, the Centers for Disease Control and Prevention’s National HIV Behavioral Surveillance Questionnaire, and other published survey items on HIV-related health information behavior [49,50]. The HIV-related health information behavior survey items selected for inclusion were based on theoretical models of information behavior, including an adapted version of the Wilson model of information behavior [51] and the Erdelez model of information encountering [52]. The survey was pilot tested on a community advisory board comprising YBMSM (data not included in the final analysis). The final survey contained 72 possible questions and took an average of 15.7 minutes to complete. In addition to sociodemographic information, the survey content included questions regarding technology ownership or use, HIV information behaviors, HIV or STI testing, HIV or STI treatment, and PrEP use. The web-based survey was administered using Qualtrics software (Qualtrics).

A total of 225 interested parties clicked on the Qualtrics link to read about the survey. Among these, 45.3% (102/225) consented to participate in the survey. Among these 102 participants, 11 (10.7%) were not eligible as they identified as female (5/102, 4.9%), 2 (1.9%) were aged above 34 years, and 4 (3.9%) reported a zip code outside of the state of North Carolina. Of the remaining 91 eligible participants, 83 (91%) completed the survey.

Descriptive statistics of the survey measures were conducted using Microsoft Excel (Microsoft Corporation) to summarize survey data, including sociodemographic characteristics and technology ownership or usage. In this paper, the reported HIV-related information behaviors included information seeking, incidental information acquisition, and information use. To assess HIV information seeking, 3 measures were used: decision to take action (eg, seeking motivators), selection of information source (eg, preference and trust), and sources of HIV information. To measure the frequency of incidental HIV information acquisition, participants were asked to respond how much times (“never,” “a little,” “some,” or “a lot”) in the past 2 years they had received HIV information in the following ways: “I learn unexpected things about HIV while browsing the Internet.” Additional response options were reflected in the data. HIV information use was measured by asking participants to identify the ways in which they applied the HIV information they acquired.

### Part II: Interviews

Between June and July 2019, 22 men who completed a survey in the first phase of the study participated in semistructured interviews using Zoom videoconference software (Zoom software citation [53]). Only survey respondents who consented to being contacted to participate in an interview were considered for selection. Purposive sampling was used to select interviewees based on the following criteria: HIV-positive diagnosis (n=9), HIV-negative and using PrEP (n=7), and HIV-negative and not using PrEP (n=6). Given that most previous studies of HIV-related information behaviors have been conducted among PLWH, the purposive sampling criteria were developed to include the perspectives and experiences of YBMSM at different points of engagement along the HIV prevention and care continuum.

Interviews, which lasted on an average for 1 hour and 16 minutes, were guided by participant responses to survey items and included questions on topics including experiences of using mobile devices and the internet to locate HIV information, incidental acquisition of HIV information while using social networking sites and geospatial dating apps, experiences acquiring HIV information offline, motivators and deterrents of HIV information acquisition, the application of HIV information, use of technologies to overcome barriers to HIV information acquisition, and their criteria for evaluating and selecting HIV information sources. All participants who completed the interview were given a US $35 Amazon e–gift card. Interviews were audio recorded, transcribed verbatim, and member checked. Each participant was assigned a pseudonym.

The interview transcripts were analyzed thematically using Dedoose software (version 8.0.35) using grounded theory methodologies [54,55]. Our thematic analysis was guided by the Wilson model of information behavior, Erdelez model of information encountering, and Kari conceptualization of information use [51,52,56]. The Wilson model of information behavior explores the following:

The totality of human behavior in relation to sources and channels of information, including both active and passive information seeking, and information use. Thus, it includes face-to-face communication with others, as well as the passive reception of information as in, for example, watching TV advertisements, without any intention to act on the information given [51].

The main constructs of the Wilson model of information behavior used for this thematic analysis were *activating mechanisms*, which are the factors motivating a decision to take action to satisfy a need for information (eg, information seeking), and the *sources and channels of information*. The Erdelez model of information encountering focuses on information acquired incidentally rather than through intentional information seeking [52]. We applied Kari conceptualization of information use as the application of a resource in some specific action [56].

Each interview transcript was read, and all texts related to HIV information acquisition and use were highlighted. The selected text was re-read and coded line-by-line to identify the initial emergent themes. Next, the most frequently reappearing initial codes were selected to begin explaining larger sections of data, and codes were condensed based on their thematic similarity. Codes were compared within and across interview text data to yield the most significant themes and provide more detailed information about HIV information acquisition and use among YBMSM.

## Results

### Internet Survey Sample

The average age of the internet sample was 29.2 (range 19-34) years. Most of the sample (70/83, 84%) consisted of men aged between 26 and 34 years. Most identified as gay (67/83, 80%), with 15% (13/83) identifying as bisexual. More than half of the sample (46/83, 55%) had less than a bachelor’s degree and were employed (62/83, 74%; Table 1). Most participants had health insurance (56/83, 67%), and 32% (27/83) reported being uninsured. The annual household income varied with one-third (34%) of the participants earning less than US $20,000 per year. A total of 43% (36/83) of participants self-reported receiving an HIV-negative test result, 41% reported living with HIV, and 15% (13/83) were unsure of their HIV status. Of the men living with HIV, 68% (23/34) were using antiretroviral therapy (ART) at the time of the study, and only 30% (11/36) of the HIV-negative men used PrEP.

**Table 1:** Internet sample demographics (N=83).

Sample demographics			Value, n (%)
**Age (years)^a^**			
	18-25	13 (15)	
	26-34	70 (84)	
**Education**			
	Less than high school	3 (3)	
	High school or GED^b^	35 (42)	
	Associate degree	8 (9)	
	Bachelor’s degree	24 (29)	
	Graduate degree	13 (15)	
**Health insurance status**			
	Insured	56 (67)	
	Uninsured	27 (32)	
**Employment**			
	Unemployed	12 (14)	
	Full-time student	9 (11)	
	Employed part time	12 (14)	
	Employed full time	50 (60)	
**Annual household income (US $)**			
	<20,000	28 (34)	
	20,000-39,000	30 (36)	
	40,000-69,000	15 (18)	
	70,000-149,000	10 (12)	
**Residence**			
	Urban	53 (64)	
	Rural	18 (21)	
	Regional city and suburban	12 (14)	
**Sexual orientation**			
	Homosexual	67 (80)	
	Bisexual	13 (15)	
	Other	3 (3)	
**HIV testing, lifetime (self-report)**			
	Never tested	6 (7)	
	Previously tested	77 (93)	
**HIV status (self-report)**			
	Positive	34 (41)	
	Negative	36 (43)	
	Unknown	13 (15)	
PrEP^c^ use (self-report)			11 (13)
ART^d^ use (self-report)			23 (28)

^a^Mean 29.2 (range 19-34) years.

^b^GED: general education development.

^c^PrEP: pre-exposure prophylaxis.

^d^ART: antiretroviral therapy.

#### Technology Ownership and Use

Participants reported significant access to multiple technological devices and frequent internet use (Table 2). Most participants (83/83, 100%) reported connecting to the internet through a cellular network on a mobile phone, with 94% (78/83) using a wireless connection. Approximately 100% (83/83) of the survey respondents who owned a mobile phone reported using the internet daily. In the web-based survey, the past use of eHealth and mHealth for HIV information was found to be high. Most men had used mobile phones or computers to seek HIV information.

**Table 2:** Technology ownership and use—internet sample (N=83).

Internet sample		Value, n (%)
**Technology ownership**		
	Smartphone	79 (95)
	Mobile phone	4 (5)
	Laptop computer	63 (76)
	Desktop computer	46 (43)
	Tablet	47 (57)
	Gaming console	58 (70)
**Internet connectivity**		
	Cellular network (3G/4G)	83 (100)
	Wi-Fi	78 (94)
	Broadband via DSL^a^, cable, or fiber optics	19 (23)
**Frequency of internet use**		
	Use internet daily on smartphone or mobile phone	83 (100)
**Past eHealth use and HIV information seeking**		
	Used mobile phone to look for HIV testing location	50 (60)
	Used mobile phone to find other HIV information	79 (95)
	Used computer to find HIV testing location	19 (23)
	Used computer to find other HIV information	60 (72)

^a^DSL: digital subscriber line.

#### HIV Information Seeking and Source Selection

Most men (28/83, 34%) in the internet sample decided to seek HIV information as they believed it would be need-to-know information for the future (Table 3). Other participants (25/83, 30%) sought HIV information to satisfy their existing information needs and some (20/83, 24%) sought HIV information following a conversation with a member of their social network (eg, a friend, family member, or romantic partner). Approximately half of the participants (42/83, 50%) preferred to acquire HIV/AIDS information from a doctor or health care provider. The internet was the second most preferred source of HIV information at 36% (30/83). The 3 most trusted sources of HIV/AIDS information were a doctor or health care provider, the internet, and libraries, with 96% (80/83), 87% (72/83), and 72% (60/83), respectively, reporting that they trust these sources “a lot” or “some.” When asked, “the most recent time you looked for information related to HIV where did you go first?”, the internet was the top choice reported by 84% (70/83) of the participants. Web-based HIV information seeking was primarily mediated by default mobile phone search engines (eg, Google, Bing, and Yahoo). Consumer health information websites (31/83, 37%), government health information websites (30%), and social media sites (17%) were the top sources of intentionally sought web-based HIV information.

**Table 3:** HIV information acquisition and use—internet sample (N=83).

Internet sample			Value, n (%)
**Decision to take action and seek HIV information**			
	Need-to-know information for the future		28 (34)
	Satisfy existing information need		25 (30)
	Following conversation with member of social network		20 (24)
	Experiencing HIV or STI^a^-like symptoms		10 (12)
**Selection of information source**			
	**Preferred sources of HIV information**		
		Doctor or health care provider	42 (50)
		Internet	30 (36)
		Community organization	7 (8)
		Friend or coworker	3 (3)
		Print media (eg, book or pamphlet)	1 (1.4)
	**Trust on sources of HIV information**		
		Doctor or health care provider	80 (96)
		Internet	72 (87)
		Libraries	60 (72)
		Newspaper or magazine	20 (24)
**HIV information seeking**			
	**Most recent source of HIV information**		
		Internet	70 (84)
		Doctor or health care provider	9 (11)
		Community organization	3 ()
		Book	1 (1)
	**Sources of HIV information in the past 2 years**		
		Internet	80 (96)
		Doctor or health care provider	41 (49)
		Print media (eg, book, pamphlet, magazine, or newspaper)	29 (5)
		Community organization	16 (19)
		Friend or coworker	11 (13)
	**Sources of web-based HIV information**		
		Consumer health information	31 (37)
		Government health information websites	25 (30)
		Social media site	14 (17)
		Community organization website	13 (16)
	**Sources of incidentally acquired HIV information**		
		Advertisements on social media sites and geospatial dating apps	54 (65)
		Postings on social media sites from web-based social ties	44 (53)
		Advertisements while browsing the web (non–social media platforms)	40 (48)
		While talking to other people	30 (36)
		While watching television or reading the news paper	27 (32)
**Social media sites and geospatial dating apps (advertisements and postings from web-based social ties)**			
	Facebook		44 (53)
	Instagram		40 (48)
	Jack’d		38 (46)
	Tinder		21 (25)
	Grindr		20 (24)
	Twitter		11 (13.2)
	Tumblr		6 (7)
	Adam4Adam		4 (5)
	YouTube		2 (2.4)
**Application of HIV information**			
	Find HIV or STI testing location		56 (67)
	Received an HIV test		53 (64)
	Begin treatment for HIV within 3 months following diagnosis (participants living with HIV)		19 (53)
	Discuss HIV status with sexual partners		46 (55)

^a^STI: sexually transmitted infection.

#### Incidental HIV Information Acquisition

Incidental HIV information acquisition was high among the internet sample (Table 3) [57,58]. Respondents (54/83, 65%) most often incidentally acquired HIV information on the web via advertisements on social media sites and geospatial dating apps, postings on social media sites from their web-based social ties (eg, people and organizations they follow; 44/83, 53%), and advertisements encountered while browsing the internet (40/83, 48%). Most participants who incidentally acquired HIV/AIDS information while using social media sites or dating apps did so via Facebook (44/83, 53%), Instagram (40/83, 48%), and the dating app Jack’d (38/83, 46%).

#### Information Use

Most men in the internet sample applied the HIV information they acquired by looking for HIV or STI testing locations (56/83, 67%) and utilizing HIV or STI testing services (53/83, 64%; Table 3). Among participants who among participants who were living with HIV (34/83), more than half (19/34, 53%) used the knowledge of their status to begin ART within 3 months of receiving a diagnosis. Approximately 55% (46/83) of the internet sample used the HIV information they acquired to discuss HIV status with their sexual partners.

### Interview Sample

#### Interview Sample Characteristics

Semistructured interviews were conducted with 22 YBMSM, aged between 22 and 33 years, who completed the web-based survey during the first phase of the study (Table 4). The interview sample included individuals residing in 9 counties in the state of North Carolina. A total of 9 men were living with HIV, 7 men were HIV-negative and using PrEP, and 6 men were HIV-negative and not using PrEP. The sample criteria were selected to reflect the varying HIV information needs and information interactions of YBMSM at different points of engagement along the HIV prevention and care continuum.

**Table 4:** Interview sample demographic characteristics (n=22).

Interview sample characteristics		Value, n (%)
**Age (years)^a^**		
	18-25	5 (23)
	26-34	17(77)
**Education**		
	Less than high school	1 (4)
	High school or GED^b^	7 (32)
	Associate degree	3 (13)
	Bachelor’s degree	6 (27)
	Graduate degree	5 (23)
**Health insurance status**		
	Private health insurance	10 (46)
	Medicaid	3 (13)
	Uninsured	9 (41)
**Employment**		
	Employed part time	6 (27)
	Employed full time	11 (50)
**Annual household income (US $)**		
	<20,000	9 (41)
	20,000-39,000	8 (36)
	40,000-69,000	5 (23)
**Residence**		
	Urban	16 (73)
	Rural	4 (18)
	Regional city and suburban	2 (9)
**Sexual orientation**		
	Homosexual	17 (77)
	Bisexual	5 (23)
**HIV testing, lifetime (self-report)**		
	Never tested	0
	Previously tested	22 (100)
**HIV status (self-report)**		
	Positive	9 (41)
	Negative	13 (59)
PrEP^c^ use (self-report)		7 (54)
ART^d^ use (self-report)		9 (100)

^a^Mean 28.8 (range 22-34) years.

^b^GED: general education development.

^c^PrEP: pre-exposure prophylaxis.

^d^ART: antiretroviral therapy.

#### The Internet as a Primary Source of HIV Information

The use of the internet and mobile technology for HIV information was high in both the web and survey samples. All men in the interview sample confirmed using a mobile phone to search for HIV information on the internet. The most common themes were convenience and the ease of accessibility that mobile phones provided for seeking HIV information from multiple sources. Most perceived the HIV information they acquired on the web to be accurate:

Mostly because they usually give me the correct answer. It’s really easy to navigate and you can find reputable sources like dot orgs or dot gov that’s really accurate, something you can believe in as a source. That’s why I trust it and then you don't have to go anywhere. Like you can pull out your phone and with quick searching, know the answer in like 10 seconds. Okay. Yep.

Preston, HIV-negative, using PrEP, 23 years

Government health information websites and health organization websites were deemed to be the most trustworthy sources of web-based HIV information. Men also valued the ability to seek HIV information privately through the internet while using a mobile phone but expressed concerns about others discovering their browsing history:

I usually use my phone and I like go into private browsing. You know like you just start typing something or if someone is using my phone I don’t want them to start typing something, then it auto fills and then I hear “hey, what’s this?”

Nathan, HIV-positive, 30 years

In the interviews, men confirmed that doctors and health care providers were their preferred and most trusted source of HIV information. Despite this preference, mobile HIV information seeking was used by men to circumvent barriers preventing them from accessing it from doctors and health care providers. The most common subthemes included discomfort communicating with health care providers about same-sex sexual behaviors and the underutilization of health care providers as an information source because of the experienced and anticipated intersectional stigma rooted in racism, homophobia, and HIV stigma in health care settings:

I didn’t tell my doctor that I was sleeping with men, and he didn’t ask. I’m already Black, and when they see me, they already treat me like I’m a statistic. I’d rather not talk to them about this stuff, because I don’t want them to treat me even worse.

Rashod, HIV-negative, not using PrEP, 22 years

#### HIV Information Use

The internet was used to seek HIV information by men in the interview sample to assess their risk of acquiring or transmitting HIV, support their skill building for HIV prevention, inform patient–doctor communication, and learn about HIV prevention and treatment options (Table 5). HIV-negative and participants living with HIV used resources to build skills to discuss their HIV or STI status and testing history with their sexual partners. One participant described his experience looking for HIV information about PrEP and eventually initiating PrEP to minimize his HIV risk once he started dating someone who was HIV-positive:

My expartner was HIV-positive. And I knew that going into that relationship, so I started to do research myself. Just looking online, browsing different online websites. And then, when I wanted to pursue Truvada, I actually spoke with a local PRIDE center.

Artez, HIV-negative, taking PrEP, 32 years

Several participants used HIV information to inform patient-doctor communication, including (1) participants *self-diagnosing* themselves before seeing a health care provider, (2) verifying HIV information they acquired on the web by speaking with a health care provider, and (3) seeking information to educate health care providers about PrEP. One participant described his experience of self-diagnosing himself before speaking with a provider because of the presence of flu-like symptoms (Table 5).

**Table 5:** HIV information use—interview sample (n=22).

Interview sample			Illustrative quotes from participants
Manage or assess risk for HIV/AIDS			“When I learned about medication resistance, it made me really on top of taking my medication. Because previously, I might go in for a sore throat and they’ll give you some antibiotics and they’ll tell you, ‘hey, make sure you take two a day for 10 days, make sure you finish it,’ and here we are two months later, and I still have like 4 pills there. So, I wasn’t always on top of it. Once I started feeling better, I’d be like, ok whatever. But learning about medication resistance particularly with HIV has def made me very much on top of taking medication as I’ve been directed to by my doctors. I have to aggressively handle my sexual health myself.” [Jerry, HIV-positive, 28 years]
**Inform patient-doctor communication**			
	Self-diagnosis	“Well, a lot of times I do a lot of self-diagnosing first just to kind of give them an idea as to what’s going on, give myself an idea of what’s going on. And to be able to explain my signs and symptoms more in detail. So that gives them a better view of what potentially could be going on. So, I went ahead and got tested just to make sure it was ok...and then if there was treatment to go along with it, it would be syphilis, the series of three shots, over the course of like six weeks...and I never wanted to go through that again, so I ask my doctor what to look for in myself and in others.” [Chris, HIV-negative, non-PrEP^a^ user, 29 years]	
	Information verification	“Whenever there is something new or a new medication or something that I hear about I will research it. And look at it. Or if it’s like has to do with different studies, I’ll look at the things that’s part of the study. And then I will ask that information of my doctor.” [Thomas, HIV-positive, 24 years]	
	Educate health care providers	“I just know I have a few friends that had experiences going to doctors, asking for PrEP or anything like that and the doctor was very closed minded or not very up-to-date on medical history in our aspects. We have to look for stuff on our own to tell them about it. I don’t like that. I still mostly trust doctors, but I’ll always think I’ll have a shred of not doubt, but like I’m gonna double check and make sure you’re telling me the best stuff. I’m going to make sure I’m with a good doctor...who is knowledgeable on whatever I need them to be knowledgeable on.” [John, HIV-negative, PrEP user, 26 years]	
Skill building for HIV prevention			“I started looking for this information because I needed help figuring out how to talk about it with other people. I’m to the point now where in the past if I got burned by somebody, I wouldn’t ask them, I would just go get treated. I was afraid of saying something to this person. But I do better now just telling people like, ‘hey I just went and got treated you might want to get tested on what’s going on.’ I was afraid to do so because the first thing you think about is them passing judgment.” [Will, HIV-negative, non-PrEP user, 27 years]
Understand HIV prevention and treatment options			“I would like look up, cause I found out there’s different strands to HIV, like it’s not just one person has HIV, like there’s levels to it. So, I’d try to Google and find information about that. And I also heard about PrEP, it was like maybe a cure or whatever I guess to try to prevent you from getting it, or lessen your chance of getting it. To be honest, my doctor had told me about it before, but I wanted to do my own research on it, so I just Googled and read up on that to find out what it’s all about.” [Patrick, HIV-negative, non-PrEP user, 32 years]

^a^PrEP: pre-exposure prophylaxis.

#### Use of Social Media for HIV Information Support Exchange

Although participants expressed displeasure with the framing and tone of advertisements on social media sites and geospatial dating apps promoting HIV prevention, they still used the sites as a space for informational support and communication with other Black MSM. Many participants reported acquiring HIV information from Black and other MSM of color who posted about their experiences living with HIV, being in a mixed HIV status relationship (serodiscordant), and the adoption of HIV risk-reduction behavior (eg, PrEP use and HIV self-testing):

I guess you can say that with me being on this medication, I do have a best friend that was also pretty much in the same boat as I am dealing with someone that was HIV positive as well. He really didn’t know much about PrEP as well, so I talked to him about it, I gave him the same information that my doctor pretty much you know gave to me. I actually pretty much introduced him to my doctor that I’m with right now, and so they’ve been on PrEP for a while now. I guess you can say that I’ve been on it, and I hadn’t received this information prior, and I was able to share it with someone else that was pretty much unaware of it as well.

Antonio, HIV-negative, using PrEP, 27 years

Unfortunately, some participants noted their diminished ability to exchange HIV information with Black and other MSM of color because of censorship policies on social media sites. When probed about where he obtained HIV information on the web, one participant noted his loss of HIV information from Black MSM on Tumblr. Other participants noted the loss of online community due to the suspension of some accounts and content removed because of censorship policies.

#### Concerns About Incidentally Acquired Web-Based HIV Information

The widespread use of social media sites and geospatial dating apps was confirmed in the interview sample. Encountering HIV information on these platforms was common. All participants expressed concerns about the framing and tone of the HIV prevention information and messages they encountered on the web via mass media HIV-prevention advertisements. Men described the framing of HIV prevention information as “judgmental” and perceived the tone of the information to be “pushy” and “fearmongering”:

The ads were overreacting, but just in a way that’s like, do this or you’ll die or do this and you definitely get, you know, you’ll definitely get syphilis...do this or you’ll end up here or there and it’s kind of stigmatizing, because I know I noticed a lot of their targeted audience where it wasn’t really straight male, and it isn’t fair to the LGBT community cause it’s almost like, okay, you’re targeting them, like making them feel like they have to get tested because they have something because they’re a part of this community versus this guy, he’s on the football team or this girl who is a cheerleader. Right. And they have, they may have something, not know it and passing it along to everyone else.

Rick, HIV-negative, non-PrEP user, 22 years

## Discussion

### Principal Findings

The purpose of this mixed methods study among YBMSM who use the internet was to explore their HIV-related health information behavior and to conduct formative work to inform the development of future, culturally tailored eHealth HIV interventions. The study found high technology ownership and use of mobile and social computing technologies to facilitate the utilization of HIV prevention and care services. Web-based HIV information was highly accessible to men in the sample through mobile phones and computers. Men in the interview sample discussed using eHealth to circumvent barriers to obtaining HIV information offline from a health care provider and for the ability to seek this information using private browsers. These findings indicate the importance of considering privacy and confidentiality when developing culturally tailored eHealth HIV interventions for YBMSM.

Men in the internet and interview sample preferred to acquire HIV information from a health care provider. However, men primarily used the internet to acquire HIV information because of barriers, including discomfort disclosing same-sex sexual behaviors to a health care provider, and intersecting stigmas experienced in health care settings. Previous studies with Black MSM have found perceptions of racism and medical mistrust to affect provider communication, health care access, and uptake of prevention services [59,60]. Black MSM have been found to be less likely to report their same-sex sexual behaviors to primary care providers compared with White MSM [57]. Prior research with young men who have sex with men (YMSM) reported barriers to communicating same-sex sexual behaviors and sexual health concerns to providers because of the fear of heterosexist bias, concerns about sexual health information being disclosed to parents, and a general belief that sexual minority youth did not receive equitable treatment in health care settings [58]. As reported in this study, the intersecting stigmas YBMSM experience related to sexuality, HIV, and racism can hinder access to HIV information from reliable and trusted sources of information [61,62]. To support engagement in HIV prevention and care, structural barriers to accessing HIV information such as racism, HIV-stigma, and homophobia must be minimized [63-65]. Future culturally tailored eHealth HIV interventions should aim to reduce intersectional stigma at the point of care and improve communication between providers and YBMSM. The development of these interventions should be community-driven. YBMSM need to be engaged in each step of the research process to identify and prioritize their needs and experiences during health care encounters. Health care providers should also receive training to provide antiracist, culturally competent care to YBMSM [66,67].

Health care providers must be equipped to provide timely recommendations on HIV prevention methods such as PrEP and HIV or STI testing [68]. In the interview sample, YBMSM shared experiences speaking with health care providers who were uninformed about PrEP and other HIV-prevention methods such as undetectable equals untransmittable (U=U; ie, a person who is HIV-positive with a consistently undetectable viral load cannot transmit HIV to a sex partner). To assist health care providers in providing timely HIV-prevention recommendations, the application of machine learning methods could be used to create an HIV risk prediction tool that identifies potential candidates for PrEP and other HIV-prevention strategies [69,70].

Previous research has found that when communicating about sensitive subjects, people may be more comfortable interacting with humans through a computer than through face-to-face interactions [71,72]. Owing to the high use of eHealth for HIV information acquisition and use among YBMSM in this sample, eHealth modalities may be especially advantageous for facilitating shared decision making between men and providers. eHealth interventions for YBMSM should aim to enhance shared decision making by increasing awareness and practical expectations of the advantages and disadvantages of prevention strategies such as PrEP and provide an environment in which they are comfortable communicating with providers [73,74]. This may help mitigate obstacles at the social, structural, and individual levels before enrolling them in health care services [75,76].

Concerns about the framing and tone of web-based, mass media HIV-prevention advertisements targeting YBMSM were raised during this study. Participants perceived the advertisements to be stigmatizing and judgmental of YBMSM. This aligns with research that found public health messaging aimed at reducing HIV disparities to be alienating [77-79]. Pleasure-centric and sex-positive framing of HIV information and messages may be helpful for promoting HIV risk-reduction behaviors among YBMSM [80]. To avoid further stigmatization of YBMSM, future eHealth HIV interventions should include community-informed, culturally tailored HIV messaging [81].

The use of social media sites for sharing and exchanging HIV information with web-based communities of Black MSM was a common theme of this study. Participants valued receiving HIV information from Black MSM who shared their experiences living with HIV, navigating serodiscordant relationships, and using PrEP and HIV self-testing kits. This aligns with the literature that found that social media sites are often used by LGBTQ individuals who may use these platforms to find information that may not be available through their offline social networks, formal education spaces, or health care providers [82,83].

In this study, it was noted that participants experienced diminished access to HIV information from Black MSM on the web because of censorship policies on social media sites such as Tumblr. These censorship policies are a direct result of FOSTA/SESTA legislation, and point to its harmful impact on minoritized communities who relied on these spaces for online social support building [84]. Participants described Tumblr as a platform where they built community and received nonstigmatizing information related to HIV. This finding supports previous research that conceptualized Tumblr as a “queer technology” and explored how the policy change pushed away communities of users, especially communities that relied on the “adult content” banned in this space for medical education and knowledge [85]. Future eHealth interventions should provide online spaces, such as forums, that promote community building and information support exchange among YBMSM.

### Limitations

This study has several limitations. First, the findings of this study are limited in their generalizability and thus do not encompass the experiences of all YBMSM, as local and individual differences are also present from this data source. Many participants living with HIV in the study were recruited from HIV clinics and community-based organizations and thus may not be reflective of the population of YBMSM in North Carolina who are out of care. The size of the quantitative sample and qualitative nature of the study also make it difficult to generalize the results to all YBMSM in the United States. Future research would benefit from a larger study sample and a more rigorously recruited study sample. However, this study does show strength over previous internet-based research with YMSM, which has traditionally been limited to mostly White participants in a single geographic region for the sample. Second, internet access familiarity with web-based technology may have influenced the decision to participate in the study. YBMSM who use a different type of electronic device to access the internet may be able to navigate websites easily and may encounter technical challenges.

Although web-based surveys and video chat platforms for qualitative data collection have been found to reduce response bias and minimize barriers for research participation (eg, financial barriers related to travel expenses or time off from work, stigma associated with research participation, and physical disabilities precluding mobility), they do not account for limited literacy. Notwithstanding these limitations, our findings represent a novel characterization of HIV information acquisition and use among YBMSM in the southeastern United States who use the internet—a topic and population that warrants further study given the high rates of HIV diagnoses among this population in this geographic region. The data from this study may provide invaluable information concerning limitations to HIV information access and the potential to use technologies to reduce social, structural, and individual-level barriers to HIV prevention and treatment among YBMSM.

## Abbreviations

ART: antiretroviral therapy
LGBTQ: lesbian, gay, bisexual, trans, and queer
mHealth: mobile health
MSM: men who have sex with men
PLWH: people living with HIV
PrEP: pre-exposure prophylaxis
STI: sexually transmitted infection
YBMSM: young Black men who have sex with men
YMSM: young men who have sex with men

## References

[1] Millett GA, Peterson JL, Flores SA, Hart TA, Jeffries WL, Wilson PA, Rourke SB, Heilig CM, Elford J, Fenton KA, Remis RS (2012). Comparisons of disparities and risks of HIV infection in black and other men who have sex with men in Canada, UK, and USA: a meta-analysis. The Lancet.

[2] Centers for Disease Control & Prevention HIV among African American gay and bisexual men.

[3] Hess KL, Hu X, Lansky A, Mermin J, Hall HI (2017). Lifetime risk of a diagnosis of HIV infection in the United States. Annals of Epidemiology.

[4] Matthews DD, Herrick AL, Coulter RWS, Friedman MR, Mills TC, Eaton LA, Wilson PA, Stall RD (2016). Running Backwards: Consequences of Current HIV Incidence Rates for the Next Generation of Black MSM in the United States. AIDS Behav.

[5] Hightow-Weidman L, LeGrand S, Choi SK, Egger J, Hurt CB, Muessig KE (2017). Exploring the HIV continuum of care among young black MSM. PLoS ONE.

[6] Maulsby C, Millett G, Lindsey K, Kelley R, Johnson K, Montoya D, Holtgrave D (2014). HIV among Black men who have sex with men (MSM) in the United States: a review of the literature. AIDS Behav.

[7] Raifman J, Dean LT, Montgomery MC, Almonte A, Arrington-Sanders R, Stein MD, Nunn AS, Sosnowy CD, Chan PA (2019). Racial and Ethnic Disparities in HIV Pre-exposure Prophylaxis Awareness Among Men Who have Sex with Men. AIDS Behav.

[8] Rebeiro PF, Ivey KS, Craig KS, Hulgan T, Huaman MA, Nash R, Raffanti S, Equakun KA, Person AK (2017). New Faces of HIV Infection: Age, Race, and Timing of Entry into HIV Care in the Southeastern United States. Journal of the International Association of Providers of AIDS Care (JIAPAC).

[9] Eaton LA, Driffin DD, Bauermeister J, Smith H, Conway-Washington C (2015). Minimal Awareness and Stalled Uptake of Pre-Exposure Prophylaxis (PrEP) Among at Risk, HIV-Negative, Black Men Who Have Sex with Men. AIDS Patient Care STDS.

[10] Rouffiac A, Whiteley L, Brown L, Mena L, Craker L, Healy M, Haubrick K (2020). A Mobile Intervention to Improve Uptake of Pre-Exposure Prophylaxis for Southern Black Men Who Have Sex With Men: Protocol for Intervention Development and Pilot Randomized Controlled Trial. JMIR Res Protoc.

[11] Brooks RA, Landovitz RJ, Regan R, Lee S, Allen VC (2015). Perceptions of and intentions to adopt HIV pre-exposure prophylaxis among black men who have sex with men in Los Angeles. Int J STD AIDS.

[12] Bauermeister J, Meanley S, Pingel E, Soler J, Harper G (2014). PrEP Awareness and Perceived Barriers Among Single Young Men who have Sex with Men. CHR.

[13] Holloway IW, Tan D, Gildner JL, Beougher SC, Pulsipher C, Montoya JA, Plant A, Leibowitz A (2017). Facilitators and Barriers to Pre-Exposure Prophylaxis Willingness Among Young Men Who Have Sex with Men Who Use Geosocial Networking Applications in California. AIDS Patient Care STDS.

[14] Nelson LE, Ajiboye W, Djiadeu P, Odhiambo AJ, Pedersen C, Ramos SR, Lofters A, Mbuagbaw L, Williams G (2020). A Web-Based Intervention to Reduce Decision Conflict Regarding HIV Pre-Exposure Prophylaxis: Protocol for a Clinical Trial. JMIR Res Protoc.

[15] Kalichman SC, Picciano JF, Roffman RA (2008). Motivation to Reduce HIV Risk Behaviors in the Context of the Information, Motivation and Behavioral Skills (IMB) Model of HIV Prevention. J Health Psychol.

[16] Fisher JD, Fisher WA, Amico KR, Harman JJ (2006). An information-motivation-behavioral skills model of adherence to antiretroviral therapy. Health Psychology.

[17] Zimmerman MS, Shaw G (2020). Health information seeking behaviour: a concept analysis. Health Info Libr J.

[18] Fidel R (2012). Human information interaction: an ecological approach to information behavior. MIT Press.

[19] Julien H, Fourie I (2015). Reflections of affect in studies of information behavior in HIV/AIDS contexts: an exploratory quantitative content analysis. Libr Inf Sci Res.

[20] Greyson Dl, Johnson Jl (2015). The role of information in health behavior: a scoping study and discussion of major public health models. J Assn Inf Sci Tec.

[21] Hogan TP, Palmer CL (2005). Information preferences and practices among people living with HIV/AIDS: results from a nationwide survey. J Med Libr Assoc.

[22] Huber JT, Cruz JM (2000). Information Needs and Information-Seeking Behaviors of HIV Positive Men and Women. Medical Reference Services Quarterly.

[23] Veinot TC, Meadowbrooke CC, Loveluck J, Hickok A, Bauermeister JA (2013). How "community" matters for how people interact with information: mixed methods study of young men who have sex with other men. J Med Internet Res.

[24] Veinot TC (2009). Interactive acquisition and sharing: Understanding the dynamics of HIV/AIDS information networks. J. Am. Soc. Inf. Sci.

[25] Siegel K, Raveis V (2016). Perceptions of Access to HIV-Related Information, Care, and Services among Infected Minority Men. Qual Health Res.

[26] Samal L, Saha S, Chander G, Korthuis PT, Sharma RK, Sharp V, Cohn J, Moore RD, Beach MC (2011). Internet Health Information Seeking Behavior and Antiretroviral Adherence in Persons Living with HIV/AIDS. AIDS Patient Care and STDs.

[27] Stonbraker S, Befus M, Lerebours Nadal L, Halpern M, Larson E (2016). Factors Associated with Health Information Seeking, Processing, and Use Among HIV Positive Adults in the Dominican Republic. AIDS Behav.

[28] Stevens R, Gilliard-Matthews S, Dunaev J, Todhunter-Reid A, Brawner B, Stewart J (2017). Social Media Use and Sexual Risk Reduction Behavior Among Minority Youth. Nursing Research.

[29] Dolcini MM, Warren J, Towner SL, Catania JA, Harper GW (2015). Information Age: Do Urban African American Youth Find Sexual Health Information Online?. Sex Res Social Policy.

[30] Jones RK, Biddlecom AE (2011). Is the Internet Filling the Sexual Health Information Gap for Teens? An Exploratory Study. Journal of Health Communication.

[31] Namuleme RK (2015). HIV/AIDS-related stigma and information behaviour: an ethnographic study in the UK. Health Info Libr J.

[32] Holloway IW, Winder TJ, Lea CH, Tan D, Boyd D, Novak D (2017). Technology Use and Preferences for Mobile Phone-Based HIV Prevention and Treatment Among Black Young Men Who Have Sex With Men: Exploratory Research. JMIR Mhealth Uhealth.

[33] Fields EL, Long A, Dangerfield DT, Morgan A, Uzzi M, Arrington-Sanders R, Jennings JM (2019). There’s an App for That: Using Geosocial Networking Apps to Access Young Black Gay, Bisexual, and other MSM at Risk for HIV. Am J Health Promot.

[34] Senn TE, Braksmajer A, Coury-Doniger P, Urban MA, Carey MP (2016). Mobile technology use and desired technology-based intervention characteristics among HIV+ Black men who have sex with men. AIDS Care.

[35] Tan JYR, Nguyen TT, Tabrisky A, Siedle-Khan R, Napoles AM (2018). Mobile Technology for Healthy Aging Among Older HIV-Positive Black Men Who Have Sex with Men: Qualitative Study. JMIR Aging.

[36] van den Berg JJ, Silverman T, Fernandez MI, Henny KD, Gaul ZJ, Sutton MY, Operario D (2018). Using eHealth to Reach Black and Hispanic Men Who Have Sex With Men Regarding Treatment as Prevention and Preexposure Prophylaxis: Protocol for a Small Randomized Controlled Trial. JMIR Res Protoc.

[37] Hightow-Weidman LB, Bauermeister JA (2020). Engagement in mHealth behavioral interventions for HIV prevention and care: making sense of the metrics. mHealth.

[38] Hightow-Weidman L, Muessig K, Claude K, Roberts J, Zlotorzynska M, Sanchez T (2020). Maximizing Digital Interventions for Youth in the Midst of Covid-19: Lessons from the Adolescent Trials Network for HIV Interventions. AIDS Behav.

[39] Turner D, Lockhart E, Marhefka SL (2019). Willingness of MSM Living with HIV to Take Part in Video-Groups: Application of the Technology Readiness and Acceptance Model. AIDS Behav.

[40] Muessig KE, LeGrand S, Horvath KJ, Bauermeister JA, Hightow-Weidman LB (2017). Recent mobile health interventions to support medication adherence among HIV-positive MSM. Curr Opin HIV AIDS.

[41] Khanna AS, Schumm P, Schneider JA (2017). Facebook network structure and awareness of preexposure prophylaxis among young men who have sex with men. Ann Epidemiol.

[42] Muessig KE, Baltierra NB, Pike EC, LeGrand S, Hightow-Weidman LB (2014). Achieving HIV risk reduction through HealthMpowerment.org, a user-driven eHealth intervention for young Black men who have sex with men and transgender women who have sex with men. Digit Cult Educ.

[43] Barry MC, Threats M, Blackburn NA, LeGrand S, Dong W, Pulley DV, Sallabank G, Harper GW, Hightow-Weidman LB, Bauermeister JA, Muessig KE (2019). “Stay strong! keep ya head up! move on! it gets better!!!!”: resilience processes in the healthMpowerment online intervention of young black gay, bisexual and other men who have sex with men. AIDS Care.

[44] Bauermeister JA, Golinkoff JM, Horvath KJ, Hightow-Weidman LB, Sullivan PS, Stephenson R (2018). A Multilevel Tailored Web App–Based Intervention for Linking Young Men Who Have Sex With Men to Quality Care (Get Connected): Protocol for a Randomized Controlled Trial. JMIR Res Protoc.

[45] Hightow-Weidman LB, LeGrand S, Muessig KE, Simmons RA, Soni K, Choi SK, Kirschke-Schwartz H, Egger JR (2019). A Randomized Trial of an Online Risk Reduction Intervention for Young Black MSM. AIDS Behav.

[46] Blackburn NA, Dong W, Threats M, Barry M, LeGrand S, Hightow-Weidman LB, Soni K, Pulley DV, Bauermeister JA, Muessig K (2021). Building community in the HIV online intervention space: lessons from the HealthMPowerment Intervention. Health Educ Behav.

[47] Threats M, Boyd DT, Diaz JE, Adebayo OW (2020). Deterrents and motivators of HIV testing among young Black men who have sex with men in North Carolina. AIDS Care.

[48] Ivankova NV (2006). Using Mixed-Methods Sequential Explanatory Design: From Theory to Practice. Field Methods.

[49] Stonbraker S, Smaldone A, Luft H, Cushman LF, Lerebours Nadal L, Halpern M, Larson E (2017). Associations between health literacy, HIV-related knowledge, and information behavior among persons living with HIV in the Dominican Republic. Public Health Nurs.

[50] Meadowbrooke CC, Veinot TC, Loveluck J, Hickok A, Bauermeister JA (2014). Information behavior and HIV testing intentions among young men at risk for HIV/AIDS. J Assn Inf Sci Tec.

[51] Wilson T (1997). Information behaviour: An interdisciplinary perspective. Information Processing & Management.

[52] Erdelez S (2004). Investigation of information encountering in the controlled research environment. Information Processing & Management.

[53] Zoom download center.

[54] Charmaz K (2014). Constructing grounded theory.

[55] Rubin H, Rubin I (2012). Qualitative interviewing: the art of hearing data.

[56] Kari J (2010). Diversity in the conceptions of information use. Information research.

[57] Petroll AE, Mosack KE (2011). Physician awareness of sexual orientation and preventive health recommendations to men who have sex with men. Sex Transm Dis.

[58] Fisher CB, Fried AL, Macapagal K, Mustanski B (2018). Patient–Provider Communication Barriers and Facilitators to HIV and STI Preventive Services for Adolescent MSM. AIDS Behav.

[59] Hausmann LRM, Hannon MJ, Kresevic DM, Hanusa BH, Kwoh CK, Ibrahim SA (2011). Impact of Perceived Discrimination in Healthcare on Patient-Provider Communication. Medical Care.

[60] Eaton LA, Driffin DD, Smith H, Conway-Washington C, White D, Cherry C (2014). Psychosocial factors related to willingness to use pre-exposure prophylaxis for HIV prevention among Black men who have sex with men attending a community event. Sex. Health.

[61] Eaton LA, Driffin DD, Kegler C, Smith H, Conway-Washington C, White D, Cherry C (2015). The Role of Stigma and Medical Mistrust in the Routine Health Care Engagement of Black Men Who Have Sex With Men. Am J Public Health.

[62] Threats M, Boyd D, Diaz J, Adebayo O (2020). Deterrents and motivators of HIV testing among young Black men who have sex with men in North Carolina. AIDS Care.

[63] Pohl A, Trill R, Li H, Nykänen P, Suomi R, Wickramasinghe N, Widén G, Zhan M (2016). Digital Health Literacy as Precondition for Sustainable and Equal Health Care - A Study Focusing the Users' Prescriptive. Building Sustainable Health Ecosystems. WIS 2016. Communications in Computer and Information Science.

[64] Gibson AN, Martin JD (2019). Re‐situating information poverty: Information marginalization and parents of individuals with disabilities. Journal of the Association for Information Science and Technology.

[65] Threats M, Brawner BM, Montgomery TM, Abrams J, Jemmott LS, Crouch P-C, Freeborn K, Kamitani E, Enah C (2021). A review of recent HIV prevention interventions and future considerations for nursing science. J Assoc Nurses AIDS Care.

[66] Quinn K, Dickson-Gomez J, Zarwell M, Pearson B, Lewis M (2018). “A Gay Man and a Doctor are Just like, a Recipe for Destruction”: How Racism and Homonegativity in Healthcare Settings Influence PrEP Uptake Among Young Black MSM. AIDS Behav.

[67] Arrington-Sanders R, Hailey-Fair K, Wirtz AL, Morgan A, Brooks D, Castillo M, Trexler C, Kwait J, Dowshen N, Galai N, Beyrer C, Celentano D (2020). Role of Structural Marginalization, HIV Stigma, and Mistrust on HIV Prevention and Treatment Among Young Black Latinx Men Who Have Sex with Men and Transgender Women: Perspectives from Youth Service Providers. AIDS Patient Care and STDs.

[68] Mutchler MG, McDavitt B, Ghani MA, Nogg K, Winder TJ, Soto JK (2015). Getting PrEPared for HIV Prevention Navigation: Young Black Gay Men Talk About HIV Prevention in the Biomedical Era. AIDS Patient Care and STDs.

[69] Marcus JL, Hurley LB, Krakower DS, Alexeeff S, Silverberg MJ, Volk JE (2019). Use of electronic health record data and machine learning to identify candidates for HIV pre-exposure prophylaxis: a modelling study. The Lancet HIV.

[70] Gilkey MB, Marcus JL, Garrell JM, Powell VE, Maloney KM, Krakower DS (2019). Using HIV Risk Prediction Tools to Identify Candidates for Pre-Exposure Prophylaxis: Perspectives from Patients and Primary Care Providers. AIDS Patient Care and STDs.

[71] Dinoia J, Schinke S, Pena J, Schwinn T (2004). Evaluation of a brief computer-mediated intervention to reduce HIV risk among early adolescent females. Journal of Adolescent Health.

[72] Evans A, Edmundson-Drane E, Harris K (2000). Computer-assisted instruction: an effective instructional method for HIV prevention education?. Journal of Adolescent Health.

[73] Stacey D, Hawker G, Dervin G, Tugwell P, Boland L, Pomey M, O'Connor Annette M, Taljaard M (2014). Decision aid for patients considering total knee arthroplasty with preference report for surgeons: a pilot randomized controlled trial. BMC Musculoskelet Disord.

[74] Jacklin S, Maskrey N, Chapman S (2018). Improving Shared Decision Making Between Patients and Clinicians: Design and Development of a Virtual Patient Simulation Tool. JMIR Med Educ.

[75] Anand T, Nitpolprasert C, Ananworanich J, Pakam C, Nonenoy S, Jantarapakde J, Sohn AH, Phanuphak P, Phanuphak N (2015). Innovative strategies using communications technologies to engage gay men and other men who have sex with men into early HIV testing and treatment in Thailand. Journal of Virus Eradication.

[76] Anand T, Nitpolprasert C, Trachunthong D, Kerr SJ, Janyam S, Linjongrat D, Hightow-Weidman LB, Phanuphak P, Ananworanich J, Phanuphak N (2017). A novel Online-to-Offline (O2O) model for pre-exposure prophylaxis and HIV testing scale up. Journal of the International AIDS Society.

[77] Drumhiller K, Murray A, Gaul Z, Aholou TM, Sutton MY, Nanin J (2017). “We Deserve Better!”: Perceptions of HIV Testing Campaigns Among Black and Latino MSM in New York City. Arch Sex Behav.

[78] Johnny L, Mitchell C (2006). “Live and Let Live”: An Analysis of HIV/AIDS-Related Stigma and Discrimination in International Campaign Posters. Journal of Health Communication.

[79] Spieldenner AR, Castro CF (2010). Education and Fear: Black and Gay in the Public Sphere of HIV Prevention. Communication Education.

[80] Dehlin JM, Stillwagon R, Pickett J, Keene L, Schneider JA (2019). #PrEP4Love: An Evaluation of a Sex-Positive HIV Prevention Campaign. JMIR Public Health Surveill.

[81] Kerr J, Ayangeakaa S, Combs R, Harris L, Sears J, Northington T, Burton K, Sterrett-Hong E, Parker K, Krigger K (2020). Community-Informed Development of a Campaign to Increase HIV Pre-exposure Prophylaxis (PrEP) Awareness Among African-American Young Adults. J Racial Ethn Health Disparities.

[82] Cho A (2017). Default publicness: Queer youth of color, social media, and being outed by the machine. New Media & Society.

[83] Duguay S Why tumblr's ban on adult content is bad for LGBTQ youth.

[84] Sawicki DA, Meffert BN, Read K, Heinz AJ (2019). Culturally competent health care for sex workers: an examination of myths that stigmatize sex-work and hinder access to care. Sex Relation Ther.

[85] Haimson OL, Dame-Griff A, Capello E, Richter Z (2019). Tumblr was a trans technology: the meaning, importance, history, and future of trans technologies. Feminist Media Studies.

